# Tardive Dysphoria With Selective-Serotonin Reuptake Inhibitors Treated Successfully With Atypical Antidepressants: A Case Series From Tertiary Hospital Setting

**DOI:** 10.7759/cureus.56684

**Published:** 2024-03-22

**Authors:** Debanjan Bhattacharjee, Sumit Kumar Mahto, Tejaswi Prithviraj H K, Surakshitha Poornima H K

**Affiliations:** 1 Psychiatry, Central Hospital Dhori, Phusro, IND; 2 Pharmacology, All India Institute of Medical Sciences, Deogarh, IND; 3 Psychiatry, Adichunchanagiri Institute of Medical Sciences, Mandya, IND; 4 Psychiatry, Sri Chamundeshwari Medical College, Hospital and Research Institute, Channapatna, IND

**Keywords:** treatment-resistant depression, selective serotonin reuptake inhibitors, antidepressant, mood disorders, tardive dysphoria

## Abstract

Tardive dysphoria (TDp) is a phenomenon characterized by the delayed onset or worsening of depressive symptoms following the discontinuation or alteration of antidepressant medications. TDp is a recently defined, under-recognized, and understudied condition. We present a series of five TDp cases exploring the diverse presentations, management strategies, and associated medical conditions. In all the cases, TDp manifested after prolonged use of selective serotonin reuptake inhibitors (SSRIs). All cases were successfully managed with atypical antidepressants. These cases offer insight into TDp, providing clinicians and researchers with examples of atypical trajectories in depressive symptomatology.

## Introduction

Tardive dysphoria (TDp) represents a distinctive and under-recognized clinical entity within the spectrum of mood disorders [1]. Depressive disorders have been extensively studied, but the phenomenon of TDp differs because of the association with prolonged antidepressant use [1]. “TDp is defined as a chronic treatment-resistant depressive state occurring in the setting of ongoing, persistent antidepressant treatment in subjects with a history of a recurrent major depressive disorder who have historically experienced an initial positive response to antidepressant medication (generally with their first exposure)” [2].

El-Mallakh et al. proposed the term TDp in 2011 [1]. It is distinct from typical mood disorders. A significant proportion of patients with treatment-resistant depression develop TDp [1]. They experience anhedonia and reduced interest, energy, and motivation; however, their function is frequently preserved, with no disturbances in appetite and self-care [1]. Generally, patients can differentiate TDp symptoms from their previous depressive episodes [1].

Although treatment resistance with prolonged antidepressant use is observed in clinical practice, TDp remains relatively understudied [1,3]. Delayed onset of depressive symptoms and loss of treatment efficacy during maintenance treatment have been noted with antidepressant regimens; therefore, medication duration and transitions have to be carefully monitored [3]. TDp poses diagnostic challenges and necessitates a nuanced approach to treatment. Guidelines or consensus regarding the diagnostic criteria and optimal management strategies for TDp are lacking. TDp is not yet listed in the Diagnostic and Statistical Manual of Mental Disorders. The neuropathology of TDp is not fully elucidated. The sparse literature on TDp emphasizes the need for additional research and guidelines to identify the symptoms of this condition. A PubMed search with the term “tardive dysphoria” gives 16 results with only three focusing on TDp [1,2,4].

Misdiagnosis or inadequate management of TDp may lead to adverse outcomes and diminished quality of life for affected individuals. Case studies can provide an understanding of the unique aspects of TDp. This case series presents five case studies of patients who manifested delayed-onset or exacerbated depressive symptoms following prolonged use of antidepressant medications. Since no case studies on TDp have been reported from India or Asia, through this case series, we aim to contribute to the evolving literature on TDp. We report the clinical nuances of the symptom onset and drugs involved, thereby paving the way for future investigations.

## Case presentation

Case 1

A 36-year-old married male presented with persistent low mood, anhedonia, fatigability, early morning awakening with worsening mood, feeling of worthlessness, decreased psychomotor activity, decreased attention and concentration, suicidal ideation, decreased libido, and alterations in sleep and appetite after four weeks of discontinuing escitalopram 20 mg. He had a history of two previous depressive episodes that positively responded to escitalopram 20 mg. He was on medication for two years.

Upon reducing the dose of escitalopram to 15 mg, the patient’s symptoms improved. The Hamilton Depression Rating Scale (HAM-D) score reduced from the initial episode score of 20 to 4. Escitalopram 15 mg was continued for almost six months. However, the depressive symptoms escalated. The HAM-D score increased to 10, prompting an escalation of escitalopram back to 20 mg, but this further exacerbated the symptoms, with the HAM-D score reaching 15. The patient reported prominent early morning worsening of mood, decreased libido, and recurrent suicidal ideation during this phase. The patient was gradually cross-tapered to mirtazapine over a period of four weeks, reaching a dose of 30 mg. Subsequently, the symptoms improved, as evidenced by a reduction in the HAM-D score to 3. The improvement maintained over an eight-month follow-up period. Among associated medical conditions, dyslipidemia was noted for a period of two years, with a total cholesterol level of 300 mg/dL noted during the last depressive episode.

Case 2

A 49-year-old married female presented with persistent low mood, anhedonia, fatigability, early morning worsening of mood, psychomotor retardation, passive death wishes, and decreased sleep and appetite. She had a stroke seven months prior to the presentation.

The patient initially responded well to sertraline 100 mg over an eight-week period, leading to a decrease in the HAM-D score from the initial episode score of 18 to 6. However, the patient reported a worsening of mood after approximately one year on sertraline. Sertraline was increased to 150 mg over four weeks, but this exacerbated the symptoms, reflected by an increase in HAM-D score to 14. Sertraline was gradually tapered and discontinued. Subsequently, the patient was started on bupropion, with the dose reaching up to 300 mg. The symptoms improved after four weeks of bupropion treatment, reflected by a reduction in the HAM-D score to 4. The improvement maintained over a one-year follow-up period. Among associated medical conditions, the patient had a history of hypertension for five years, for which the patient took antihypertensive medications irregularly.

Case 3

A 36-year-old married male presented with excessive worry about multiple activities, difficulty in controlling worry, restlessness, fatigability, decreased attention and concentration, muscle tension, and decreased sleep. The primary manifestation was suggestive of generalized anxiety disorder (GAD) with the patient showing a Hamilton Anxiety Rating Scale (HAM-A) score of 25.

The patient initially responded well to escitalopram 15 mg, wherein the HAM-A score reduced from 25 to 4 over a four-week period. However, the patient reported a decline in mood, anhedonia, fatigability, decreased libido, and passive death wishes after approximately six months on escitalopram 15 mg. Additionally, anxiety symptoms worsened. Escitalopram was increased to 20 mg over four weeks, but this further exacerbated the symptoms, with HAM-D and HAM-A scores reaching 22 and 20, respectively. Escitalopram was gradually cross-tapered with mirtazapine. Over a four-week period with mirtazapine 30 mg, the symptoms improved, with both HAM-D and HAM-A scores reducing to 3 and 4, respectively. The improvement maintained over a six-month follow-up period.

Case 4

A 50-year-old married female presented with persistent low mood, anhedonia, fatigability, early morning awakening with worsening mood, feeling of worthlessness, decreased psychomotor activity, decreased attention and concentration, suicidal ideation, and decreased sleep and appetite. The patient positively responded to fluoxetine 40 mg over a six-week period, wherein the HAM-D score reduced from the recent episode score of 22 to 2. However, the depressive symptoms subsequently worsened after approximately one year on fluoxetine 40 mg and escitalopram 15 mg, with an increase in HAM-D score to 12. Fluoxetine was increased to 60 mg resulting in further deterioration, with the HAM-D score reaching 16. The patient reported a prominent early morning worsening of mood, marked decreased appetite, and recurrent suicidal ideation during this phase. Fluoxetine was gradually cross-tapered with mirtazapine over an eight-week period, finally reaching a dose of 45 mg. Subsequently, the symptoms improved, with the HAM-D score reducing to 2. The improvement maintained over a six-month follow-up period.

The patient had associated medical conditions: Sturge-Weber syndrome and a history of hypertension for 10 years managed with regular antihypertensive medications. As per the medical records, she had tonic-clonic seizures many years back for which she is on carbamazepine 300 mg twice daily and has had no episode of seizure for the last 15 years.

Case 5

A 35-year-old married male presented with persistent low mood, anhedonia, fatigability, feelings of worthlessness and helplessness, decreased psychomotor activity, decreased attention and concentration, recurrent suicidal ideation, decreased libido, and decreased sleep and appetite for four weeks, while on sertraline 150 mg for approximately one year. The patient had a history of two previous depressive episodes. The patient positively responded to escitalopram 20 mg after the first episode. After the second episode, the patient responded to fluoxetine 40 mg but did not show complete improvement of symptoms and was started on sertraline 150 mg with positive outcomes.

After the recurrence of symptoms, sertraline was changed to mirtazapine 30 mg for four weeks without any improvement. The patient was then started on bupropion, with the dose reaching up to 300 mg for four weeks. Subsequently, the symptoms improved, reflected by a reduction in the HAM-D score from 24 to 4. The improvement maintained over a six-month follow-up period. Among associated medical conditions, the patient had dyslipidemia for a period of three years, with a total cholesterol level of 350 mg/dL noted during the last depressive episode. Additionally, the patient had a history of hypertension for one year, which was managed with antihypertensive medications.

Diagnostic criteria

All patients were diagnosed with TDp based on the criteria proposed by El-Mallakh et al. [1]. In all cases, most of the patient symptoms and characteristics met the proposed criteria for TDp, but a few did not (Table 1). However, all patients were diagnosed with TDp because they showed distinct dysphoric symptoms after prolonged exposure to selective serotonin reuptake inhibitors (SSRIs).

**Table 1:** Symptoms and diagnosis based on the proposed criteria for TDp. SSRI, selective serotonin reuptake inhibitor; TDp, tardive dysphoria.

Parameters	Case 1	Case 2	Case 3	Case 4	Case 5
Symptoms					
Onset	Insidious	Insidious	Insidious	Insidious	Insidious
Course	Episodic	Continuous	Continuous	Continuous	Episodic
Duration	2 years	6 months	6 months	1 year	4 years
Proposed criteria for TDp					
Prolonged (at least 1 year) exposure to a therapeutic dose of SSRI	No 6-month escitalopram	Yes 1-year sertraline	No 6-month escitalopram	Yes 1-year fluoxetine + escitalopram	Yes 1-year sertraline
Onset of a chronic or continuous dysphoric state while receiving a therapeutic dose of SSRI; the dysphoric state lacks the episodic nature of major depressive disorder	No	Yes	Yes	Yes	No
Return to baseline depressive state 4-6 weeks post-antidepressant withdrawal	Yes	Yes	Yes	Yes	Yes
Delayed and gradual improvement (after several months)	No (1 month)	No (1 month)	No (1 month)	Yes (2 months)	No (1 month)
At least 7 of the following symptoms or characteristics are present or absent					
Depressed or dysphoric mood	Yes	Yes	Yes	Yes	Yes
Reduced motivation	Yes	Yes	Yes	Yes	Yes
Reduced interest	Yes	Yes	Yes	Yes	Yes
Reduced energy	Yes	Yes	Yes	Yes	Yes
Reduced pleasure	Yes	Yes	Yes	Yes	Yes
Sleep disturbance with common middle insomnia	Yes	Yes	Yes	Yes	Yes
Mood or affective lability	Yes	Yes	Yes	Yes	Yes
Irritability	Yes	Yes	Yes	Yes	Yes
No or minimal disturbance in appetite	No	No	Yes	No	No
No or minimal disturbance in self-care	Yes	Yes	Yes	Yes	Yes

## Discussion

The presented case series sheds light on the complex and challenging landscape of TDp. While TDp is a phenomenon characterized by antidepressant-induced dysphoric symptoms due to prolonged exposure to antidepressants, all the cases presented had SSRI-induced dysphoric symptoms. The cases focus on the interplay between depressive symptoms and medication responsiveness. We also present associated comorbid medical conditions along with the varied presentations of the cases.

TDp occurs due to prolonged antidepressant use, particularly SSRIs [1]. The period and symptoms of antidepressant withdrawal are not considered as part of TDp [1]. In TDp, the gradual improvement in depressive symptoms after discontinuation or change of antidepressants may not represent full remission [1]. The cases illustrate the diverse responses to antidepressant medications, particularly SSRIs including escitalopram and fluoxetine. There is no definitive treatment for TDp; however, atypical antidepressants can be tried, which we did. The cases were resolved with atypical antidepressants like mirtazapine and bupropion. Patients’ responses to treatments vary emphasizing the importance of individualized approaches to treatment regimens including the duration of treatment.

Case 1 presents a classic case of TDp, wherein the patient had exacerbated symptoms upon prolonged use of escitalopram 20 mg, which reduced intermittently upon reduction in dose to escitalopram 15 mg but exacerbated further. The patient from case 2 had a history of stroke, which may have contributed to the psychiatric manifestations. Case 3 presented the challenge of managing comorbid anxiety and depression, emphasizing the importance of selecting appropriate interventions based on the evolving symptomatology. Case 4 highlights the challenge of managing depression in the context of complex medical comorbidities. The patient has Sturge-Weber syndrome, which introduces additional challenges in the treatment of TDp. The patient was on carbamazepine 300 mg twice daily, and drug-drug interactions should be considered in the management of depressive symptoms. Case 5 presented the complexity of managing recurrent depressive episodes and the importance of selecting interventions considering the concurrent medical conditions of dyslipidemia and hypertension. The cases do not fit into the proposed criteria for TDp; however, we suggest changes in the timelines mentioned in the proposed criteria to include certain patients who do show depressive symptoms after prolonged use of SSRIs, so that patients with TDp are diagnosed early for appropriate management.

In all cases, the patients showed an initial positive response to SSRI, a subsequent recurrence while prolonged use of the SSRI, and the ultimate success with atypical antidepressants. The patients’ sustained improvement over the follow-up period contributes valuable insights into the long-term management of cases with treatment-resistant depression. It also questions the current practice of prolonged treatment of depression with SSRIs [3]. This may not be applicable to antidepressant discontinuation in other conditions. For example, antidepressant discontinuation in patients with migraine is significantly associated with an increased risk of depression, but not TDp [5]. However, in the psychiatric setting, antidepressants are hypothesized to have a pro-depressant effect [6].

The cases also present successful outcomes observed with cross-tapering strategies and transitioning between different classes of antidepressants. The presence of comorbid medical conditions such as Sturge-Weber syndrome, hypertension, and dyslipidemia further complicates clinical management due to drug-drug interactions and drug-disease interactions. Integration of psychiatric and medical management is crucial for comprehensive care.

While this case series provides valuable clinical insights, the absence of a standardized diagnostic criterion for TDp introduces potential biases and limits the interpretation of the symptoms. Further research, including neurobiological investigations, is warranted to deepen our understanding of TDp and refine treatment guidelines. The relationship between the serotonin transporter gene, risk for depression, and response to serotonergic antidepressants is currently under study in a randomized clinical trial [6].

## Conclusions

This case series adds to the literature on TDp and paves the way for future investigations. It emphasizes the need for personalized and adaptable treatment strategies, considering factors such as medication response, comorbidities, and the evolving nature of symptoms. The successful outcomes observed with different atypical antidepressants, including mirtazapine and bupropion, highlight the necessity of changing treatment strategies and question the prolonged use of SSRIs for treating episodic depression. The unique presentation of TDp challenges traditional concepts of depressive disorders, and more such studies are needed to refine our understanding of its etiology, clinical features, disease trajectory, and management strategies.

## References

[1] El-Mallakh RS, Gao Y, Briscoe BT, Roberts RJ (2011). Antidepressant-induced tardive dysphoria. Psychother Psychosom.

[2] El-Mallakh RS, Roberts RJ, Swann A, Rutherford PR, Surja A (2011). Tardive dysphoria: antidepressant-induced chronic depression. Ir J Psychol Med.

[3] Fava GA (2020). May antidepressant drugs worsen the conditions they are supposed to treat? The clinical foundations of the oppositional model of tolerance. Ther Adv Psychopharmacol.

[4] El-Mallakh RS, Gao Y, Jeannie Roberts R (2011). Tardive dysphoria: the role of long term antidepressant use in-inducing chronic depression. Med Hypotheses.

[5] Kious BM, Bakian AV (2020). Evidence of new-onset depression among persons with migraine after discontinuing antidepressants. Psychiatry Res.

[6] Ali ZA, Nuss S, El-Mallakh RS (2019). Antidepressant discontinuation in treatment resistant depression. Contemp Clin Trials Commun.

